# Age-dependent nasal immune responses in non-hospitalized bronchiolitis children

**DOI:** 10.3389/fimmu.2022.1011607

**Published:** 2022-12-06

**Authors:** Isabel Cortegano, Mercedes Rodríguez, Susana Hernángómez, Alejandro Arrabal, Carlos Garcia-Vao, Javier Rodríguez, Sandra Fernández, Juncal Díaz, Belén de la Rosa, Beatriz Solís, Cristina Arribas, Felipe Garrido, Angel Zaballos, Sergio Roa, Victoria López, Maria-Luisa Gaspar, Belén de Andrés

**Affiliations:** ^1^ Immunobiology Department, Centro Nacional de Microbiología (CNM), Instituto de Salud Carlos III (ISCIII), Madrid, Spain; ^2^ Pediatrics Department, Hospital El Tajo, Aranjuez, Madrid, Spain; ^3^ Pediatrics Department, Atención Primaria Galapagar, Madrid, Spain; ^4^ Pediatrics Department, Hospital Puerta de Hierro, Madrid, Spain; ^5^ Pediatrics Department, Clínica Universitaria de Navarra, Madrid, Spain; ^6^ Genomics Central Core, Instituto de Salud Carlos III (ISCIII), Madrid, Spain; ^7^ Biochemistry and Genetics Department, Universidad de Navarra, Pamplona, Spain; ^8^ Chronic Disease Programme Unidad de Investigación de Enfermedades Crónicas (UFIEC), Instituto de Salud Carlos III (ISCIII), Madrid, Spain

**Keywords:** nasal lavage fluid (NLF), cytokines, immunoglobulins, B lymphocytes, monocytes, neutrophils, bronchiolitis, RSV

## Abstract

Bronchiolitis in children is associated with significant rates of morbidity and mortality. Many studies have been performed using samples from hospitalized bronchiolitis patients, but little is known about the immunological responses from infants suffering from mild/moderate bronchiolitis that do not require hospitalization. We have studied a collection of nasal lavage fluid (NLF) samples from outpatient bronchiolitis children as a novel strategy to unravel local humoral and cellular responses, which are not fully characterized. The children were age-stratified in three groups, two of them (GI under 2-months, GII between 2-4 months) presenting a first episode of bronchiolitis, and GIII (between 4 months and 2 years) with recurrent respiratory infections. Here we show that elevated levels of pro-inflammatory cytokines (IL1β, IL6, TNFα, IL18, IL23), regulatory cytokines (IL10, IL17A) and IFNγ were found in the three bronchiolitis cohorts. However, little or no change was observed for IL33 and MCP1, at difference to previous results from bronchiolitis hospitalized patients. Furthermore, our results show a tendency to IL1β, IL6, IL18 and TNFα increased levels in children with mild pattern of symptom severity and in those in which non RSV respiratory virus were detected compared to RSV+ samples. By contrast, no such differences were found based on gender distribution. Bronchiolitis NLFs contained more IgM, IgG1, IgG3 IgG4 and IgA than NLF from their age-matched healthy controls. NLF from bronchiolitis children predominantly contained neutrophils, and also low frequency of monocytes and few CD4^+^ and CD8^+^ T cells. NLF from infants older than 4-months contained more intermediate monocytes and B cell subsets, including naïve and memory cells. BCR repertoire analysis of NLF samples showed a biased VH1 usage in IgM repertoires, with low levels of somatic hypermutation. Strikingly, algorithmic studies of the mutation profiles, denoted antigenic selection on IgA-NLF repertoires. Our results support the use of NLF samples to analyze immune responses and may have therapeutic implications.

## Introduction

Bronchiolitis is a common respiratory pathology with significant rates of morbidity and mortality that predominantly affects children. The clinical manifestations of this condition involve acute inflammation of the upper and lower respiratory airways, which requires treatment to overcome the breathing difficulties that arise and to clear the lung mucus (1). Respiratory syncytial virus (RSV) infection of the lower respiratory tract is the leading cause of bronchiolitis worldwide, although metapneumovirus (MPV), influenza (FLU), adenovirus (ADENO), rhinovirus (RHN), bocavirus and parainfluenza virus (PFLU-1) may also be involved in this pathology (2–4).

Mucosal secretion of antibodies, cytokines and antimicrobial proteins shapes the effective barrier of epidermal surfaces and protects the respiratory tree against airborne pathogens and foreign proteins. Pathological RSV-dependent lung inflammation is the result of a complex cascade of events in the respiratory tract, involving the activation and recruitment of epithelial and immunocompetent cells, together with the secretion of pro-inflammatory cytokines and chemokines (5, 6). In conjunction, these events result in epithelial and ciliary destruction, increased mucus secretion, bronchial obstruction and air trapping (1, 5, 7). After RSV infection, a massive infiltration of neutrophils (Nϕs) has been detected in bronchoalveolar lavage (BAL) samples from pediatric patients (6, 8). These Nϕs can limit virus replication when activated and spread by releasing soluble mediators, and that regulate the immune response by interacting with CD8^+^ T cells, NK dendritic cells and B cells (9–11). However, activation/degranulation of Nϕs can also damage the immature lung parenchyma during infanthood and may promote the onset of asthma (8). Severe RSV infection also produces effective monocyte mobilization, reducing the classical and non-classical monocytes in the peripheral blood and also impairing IFNγ and IL12 release (12, 13). T regulatory cells (Tregs) are also involved in the immune response of RSV-infected infants, their numbers decreasing in peripheral blood (14). Furthermore, mucosal invariant T cells (MAIT), NK, NKT and Tγδ cells have been involved in the production of different cytokines under infection pathologies (15–17). In the case of hospitalized infants under 3 mo of age, severe bronchiolitis is frequently correlated with the presence of IL10-secreting B cells (Bregs) in nasopharyngeal aspirates, dampening Th1 cytokine production (18).

Most of these studies were performed on samples from hospitalized children. NLF samples may constitute an efficient tool to study the immunological responses to respiratory infections, as a non-invasive approach to trace *in situ* the immunological features in outpatient children up to 2-years-old, where access to blood samples is always difficult. Here we studied NLF from non-hospitalized age-stratified children suffering a mild/moderate bronchiolitis to assess the cellular and molecular dynamics of their immune responses where there is few available information. A comprehensive characterization of the cytokine profile, antibody content, cell composition and the B lymphocyte immunoglobulin heavy chain (IgH) repertoires of these NLF samples, revealed a pronounced pro-inflammatory cytokine profile in children with bronchiolitis. In addition, there was a significant enrichment of immunocompetent CD45^+^ cells, including both myeloid and lymphoid cells. Whereas Nϕs, inflammatory monocytes and CD4^+^ and CD8^+^ T cells were always present in bronchiolitis NLF, B cell recruitment was delayed, with overall evidence of an immature humoral response and with B cell subsets present in children older than 2 mo. This integrated characterization of the humoral and cellular responses in a non-invasive biofluid obtained from newborns and infants, showed significant age-dependent maturational changes in the nasal immune system.

## Material and methods

### Sample collection

NLF samples were obtained from infants on their first visit to the pediatrician (between November-March 2018-2019) after an episode of acute onset expiratory dyspnea, wheezing, respiratory distress and poor feeding for 24-72 hours, suspicious of viral respiratory illness that did not require hospitalization. The inclusion criteria were infants with their first respiratory infection and without any prior medical treatment, with clinical symptoms stratified as Group I (infants under 2-mo-old) or Group II (infants between 2- and 4-mo-old), the peak incidence for bronchiolitis being detected in those groups (19). We also included infants from 5- to 24-mo-old with recurrent respiratory infections in a third group (Group III) treated with glucocorticoid and leukotrien blockers. The modified Tal score was used to assess bronchiolitis severity (20). The infant pathological status was also stratified on the basis of the score as mild (score 0 to 5) and moderate (score >5-8). The exclusion criteria for this study were a diagnosis of any other infection, medical treatment or an incomplete program of vaccination. Control samples were obtained from infants with no respiratory conditions on routine visits to their pediatrician. The clinical data for each group studied are summarized in **Table 1**.

**Table 1:** Clinical data.

	Bronchiolitis			Controls			p
	GI	GII	GIII	GI	GII	GIII	
**n**	16	18	19	12	16	18	
**Age (days)** **Median ± SEM**	32.3 ± 7	99 ± 8.3	332 ± 49	37.1 ± 7	100 ± 7.5	512 ± 38	ns
**Gender M/F**	10/6	11/7	12/7	7/5	8/8	10/8	ns
**Weight at birth (Kg)** **Median ± SEM (IQR)**	3.21 ± 0.09 (2.9-3.6)	3.40 ± 0.09 (2.6-3.8)	3.32 ± 0.12 (3-3.7)	3.35 ± 0.14 (3.0-3.6)	3.22 ± 0.12 (2.7-3.5)	3.3 ± 0.11 (3.1-3.5)	ns
**Weeks of gestation** **Median ± SEM (IQR)**	39.06 ± 0.27 (38.2-40)	39.42 ± 0.30 (38.6-40)	39.38 ± 0.32 (38-40.2)	39 ± 0.27 (38-40)	39.33 ± 0.28 (38-40)	39.7 ± 0.3 (38-41)	ns
**Feeding^1^**	7/9	10/8	13/6	10/2	11/5	14/4	0.032^2^
**Days of symptoms** **Mean ± SEM**	3.47 ± 0.54	2.67 ± 0.31	2.20 ± 0.30	NA	NA	NA	0.0418^3^
**Modified Tal score** **Mild/Moderate (%)**	11/5 (69%)	16/2 (88%)	12/7 (63%)	NA	NA	NA	ns^4^
**Viral content in supernatant**							
**Virus+** **(%)**	9 (56%)	7 (38%)	13 (68%)	0 (0%)	1 (6.2%)	5 (27%)	ns^4^ 0.0001^5^
**RSV**	7	4	5	0	0	0	NA
**MPV**	0	0	0	0	0	0	NA
**FLU**	1	0	2	0	0	0	NA
**ADENO**	0	1	0	0	0	1	NA
**RHINO**	0	0	4	0	1	4	NA
**Coinfections**	1^6^	2^7,8^	2^7,9^	0	0	0	NA

Symptoms: pulmonary distress, runny nose, cough, wheezing. All patients have followed the vaccination program and their neonatal health is normal (no chronic pulmonary pathology, no cystic fibrosis, no bronchopulmonary dysplasia, no congenital lung malformations, no cardiopathies). Data were analyzed using non-parametric Kruskal-Wallis, Mann-Whitney for intragroup analysis and Chi-square for categorical comparisons. ns, represents comparisons between age-matched cohorts.

^1^Feeding (maternal/partial maternal with artificial). ^2^Chi-square GI Bronchiolitis/Control. ^3^Non-parametric Kruskal-Wallis between Bronchiolitis GI and GIII.^4^ Chi-square between Bronchiolitis groups.^5^Chi-square between Bronchiolitis/Control. Coinfections:^6^FLU, RHINO; ^7^RSV, MPV;^8^ADENO, RHINO; ^9^RSV, ADENO.

The NLF were obtained as described previously (21). Briefly, infants were seated upright with the head bent down. A syringe loaded with 2 mL of saline solution (at room temperature) was used to irrigate one nasal cavity, and 1 mL of this solution was collected on the opposite nasal fossa. The manipulation is rapid, easy to perform and well tolerated by the children. The NLF were kept at 4°C for less than 24 hr before processing. They were centrifuged at 110 g for 5 min at 4°C (Eppendorf MiniSpin, Merck Darmstadt, Germany) to separate the cellular content from the supernatant as described (21). The supernatants were aliquoted and frozen at -80 °C, whereas the cells were separated in two aliquots, one as pellet to perform next generation sequencing (NGS) analyses and the other as cell suspension analyzed immediately by flow cytometry. Control blood samples were obtained from adult donors. Peripheral blood mononuclear cells (PBMCs) were isolated by centrifugation through Ficoll-Paque™ gradient (GE Healthcare, Connecticut, EEU). NLF and PBMC cell suspensions were washed and counted with the trypan blue dye.

### Virus detection in NLF

RSV was initially determined in NLF by the pediatricians using a rapid RSV test (Quidel^®^, San Diego, CA, USA). This test detects viral fusion protein, with a sensitivity of 83% for NLF samples. In addition, viral nucleic acids were extracted from 200 µL of the NLF supernatants using NUCLISENS^®^ EASYMAG^®^ (bioMérieux, Lyon, France) according to the manufacturer´s instructions. PCR multiplex for 21 types and subtypes of respiratory viruses were performed using the CLART1^®^ Fast PnenumoVir platform (Genomica, Madrid, Spain) including RSV, MPV, FLU, ADENO and RHN. The positive correlation between the rapid RSV testing and laboratory RSV-PCR detection was over 95% (data not shown).

### Cytometric bead array (CBA) determination of cytokine and IgG subclasses in NLF

A 13-multiplex cytometric CBA (IL1β, IFNα2, IFNγ, TNFα, MCP1/CCL2, IL6, IL8/CXCL8, IL10, IL12p70, IL17A, IL18, IL23 and IL33: Biolegend, CA, USA), and human IgG isotype bead array (IgG1, IgG2, IgG3 and IgG4: Biolegend) were used to quantify the cytokines and IgG isotypes in NLF samples. The determinations were performed in duplicate according to the manufacturer´s instructions. Samples were run on a CANTO I (BD Biosciences, San José, CA, USA) cytometer and the calibration curves were above r^2^ 0.97.

### Immunoglobulin (Ig) determination by ELISA

IgM, IgGs and IgA were measured in the NLF supernatants by ELISA. Briefly, 96-well plates (Nunc, Rochester NJ, USA) were coated with unlabeled goat-anti human Igs (10 µg/ml: Southern Biotechnology Birmingham AL, USA) and they were then blocked with Phosphate buffered saline (PBS, BioWhittaker, Lonza Group Basilea Switzerland) + 0.5% gelatin. The supernatants were thawed and serial dilutions in the same buffer were added to the wells in duplicate. The plates were then incubated with biotinylated goat anti-human-IgM, -IgG or -IgA (Southern Biotechnology), and subsequently with streptavidin-peroxidase (Southern Biotechnology). The ELISA plates were revealed with 0.5M O-phenylenediamine (Sigma-Aldrich St. Louis MI, USA) and the reaction was stopped with 3N SO_4_H_2_. OD values were obtained at 450 nm and standard curves were generated using purified myeloma proteins for human IgM, IgA (Southern Biotechnology) and IgG1 (a generous gift from Dr E. Fernández, Hospital La Princesa, Madrid). The Ig concentrations were calculated using the GraphPad Prism 5.0 software.

### Flow cytometry and antibodies

Single cell suspensions were prepared in staining buffer (SB, PBS supplemented with 2.5% heat inactivated Fetal Calf’s Serum, Gibco, Waltham, MA, USA). The cells were stained with the following monoclonal antibodies: APC/Cy7-labeled anti-CD45 (clone HI30) and anti-CD27 (clone LG.3A10); PE/Cy7 anti-CD4 (clone OKT4) and anti-CD43 (clone CD43-10G7); PE anti-CD16 (clone 3G8); Alexa Fluor 488 anti-CD20 (2H7) and anti-CD14 (clone HCD14); APC anti-CD8a (clone HIT8a); Alexa Fluor 700 anti-CD19 (HIB19); PerCP/Cy5.5 anti-CD70 (clone 113-16); Alexa Fluor 647 anti-IgD (clone IA6-2); BV605 anti-CD5 (clone L17F12). All antibodies were from BioLegend (San Diego, CA, USA) except the anti-CD16 (Immunostep, Salamanca, Spain). Irrelevant isotype-matched monoclonal antibodies (BioLegend) were used as controls. Cells were fixed for 15 min at RT in 2% paraformaldehyde in SB. The cell viability was determined with the LIVE/DEAD Fixable Violet Dead Cell Stain Kit violet-510 kit (Invitrogen, Carlsbad CA, USA). The cells were analyzed in a LRS Fortessa X-20 (BD Biosciences) cytometer, using the FlowJo v10.7.1 (TreeStar, Ashland, OR, USA) and DIVA v8.0 (BD Biosciences) software packages. The gating strategy is described for a representative NLF sample in **Figure S2A**. Briefly, after gating out low FSC channels (dump), doublets (SSC-H/SSC-W and FSC-H/FSC-W) and dead cells were excluded. The anti-CD45 antibody was used to identify immune cells.

### Cell subpopulations determined by t-SNE analysis

Raw flow cytometry files were pre-processed in FlowJo v10.7.1, and live CD20^+^ B cells were gated and exported as single FCS files that included compensated parameters. These processed FCS files were submitted in FlowJo to the R package plugin DownsampleV3 (to reduce the populations to fixed number of 1,000 cells from each sample). The reduced populations were exported again as single FCS files and then were merged as a unique file for PBMCs (n = 3), NLF samples from Group II (n = 5) and Group III (n = 5) by using the concatenate tool of FlowJo. The t-SNE analysis was then performed with default parameters, including 1,000 iterations and a perplexity parameter of 30, using IgD and CD27 as markers. The t-SNE maps were generated from Ad-PBMCs and NLF samples from Group II and Group III (22) which were displayed to identify IgD^+^CD27^-^ naïve B cells, IgD^-^CD27^-^ DN B cells (defined as atypical memory B cells) and CD27^+^ memory B cells (IgD^-^, IgD^+^). B1 cells were defined as CD19^+^CD27^+^CD70^-/-^CD43^+^ CD5^+/-^ (18, 23) and they analyzed in a separate panel.

### Quantitative PCR and Ig-NGS on NLF samples

Total RNA was extracted from the NLF cell pellets with the RNeasy Micro kit (Qiagen, Hilden, Germany), according to the manufacturer´s instructions. Oligo (dT) primed cDNAs were then prepared with AMV reverse transcriptase (Promega) at 42 °C for 1 h, and subjected to RT-qPCR with specific primers (**Table S1**). The SsoFast Eva Green^®^ Supermix (Bio-Rad Hercules, CA, USA) was used for Pax5 and Glyceraldehyde 3-phosphate dehydrogenase (GAPDH) on a CFX96 Real-Time System (Bio-Rad) performed in duplicates. The Bio-Rad CFX Manager software was used to obtain the C_T_ of each reaction (24). Data were normalized to GAPDH mRNA in each sample, and the results expressed as the 2^-ΔΔCT^ referred to adult PBMC. To analyze the VDJ-C rearrangements in B cells from the NLF, the cDNA obtained was PCR amplified with the 1U Supreme NZYTaq II DNA polymerase (NZYTech, Lisboa, Portugal) in a PTC-200 DNA Engine cycler (Bio-Rad). Rearranged alleles were amplified using VH primers covering the IGHV1 to IGHV6 families, and CH-specific primers (**Table S1**) in separate tubes, all including multiple identifier sequences (MID) as described previously (25) using the MiSeq sequencer. Amplicons were prepared with the Nextera XT Index v2 kit (Illumina Inc, San Diego, CA, USA), as indicated by the manufacturer, purifying the PCR amplicons with epMotion 5075 (Eppendorf) using Agencourt AmPure XP magnetic beads (Eppendorf). The amplicons were quantified by Quantifluor One dsDNA System in a Quantus Fluorimeter (Promega) and the samples were pooled in equal quantities for sequencing with the MiSeq Reagent kit v2 (Illumina).

### Bioinformatics

Libraries were obtained by 500 bp paired-end sequencing on the MiSeq platform (Illumina). The forward and reverse reads were pre-processed with VDJPipe (26, 27) (merging sequences by filtering to minimum average quality score of 35, maximum homopolymer of 20 and collapsing in files with total fasta sequences) (26, 27) using the VDJ-server Release 1.1.2 (https://vdjserver.org/). Successfully paired sequences were sent to IMGT/HighV-Quest version 3.4.17 (28) for annotation of the CDR3 and full-length VDJ regions. The IMGT output files were analyzed in ARGalaxy (29). Only complete productive sequences, without ambiguous bases, or sequences present at least twice, were included in the analysis as a single sequence. As few productive sequences were obtained in some samples, only those containing more than 15 different sequences were used to assess the individual IgH repertoires. The SHM & CSR pipeline was used to analyze the SHM patterns, as well as antigen selection (including BASELINe (30)) and clonality. Starting from the IMGT output obtained, CDR3 graphics were obtained using the R package ggplot2.Alakazam (27). Lineage trees were created based on a minimal substitution model, with the multiple alignments achieved with MUSCLE (31), curated with Gblocks (32) and tree inference achieved with PhyML 3 (33). The tree Netwick display was obtained using iTool v.4 (34).

### Statistical analyses

The data are presented as the means ± SEM or the median ± Q1 and Q3 interquartile range (IQR). All statistical analyses were performed using GraphPad Prism 8.0 software after testing the normality of the data distributions with the Kolmogorov-Smirnov and D`Agostino-Pearson tests. The data were compared using Mann-Whitney test for non-parametric data and for parametric data using two-tailed unpaired Student`s *t*-tests with Welch´s correction and ANOVA for multiple comparisons. Categorical data comparisons were performed using Chi-square test. Two-tailed Pearson and Spearman correlation coefficients were calculated with 95% confidence interval.

## Results

### Experimental design, clinical parameters and determination of respiratory viruses in NLF samples

**Figure 1:**
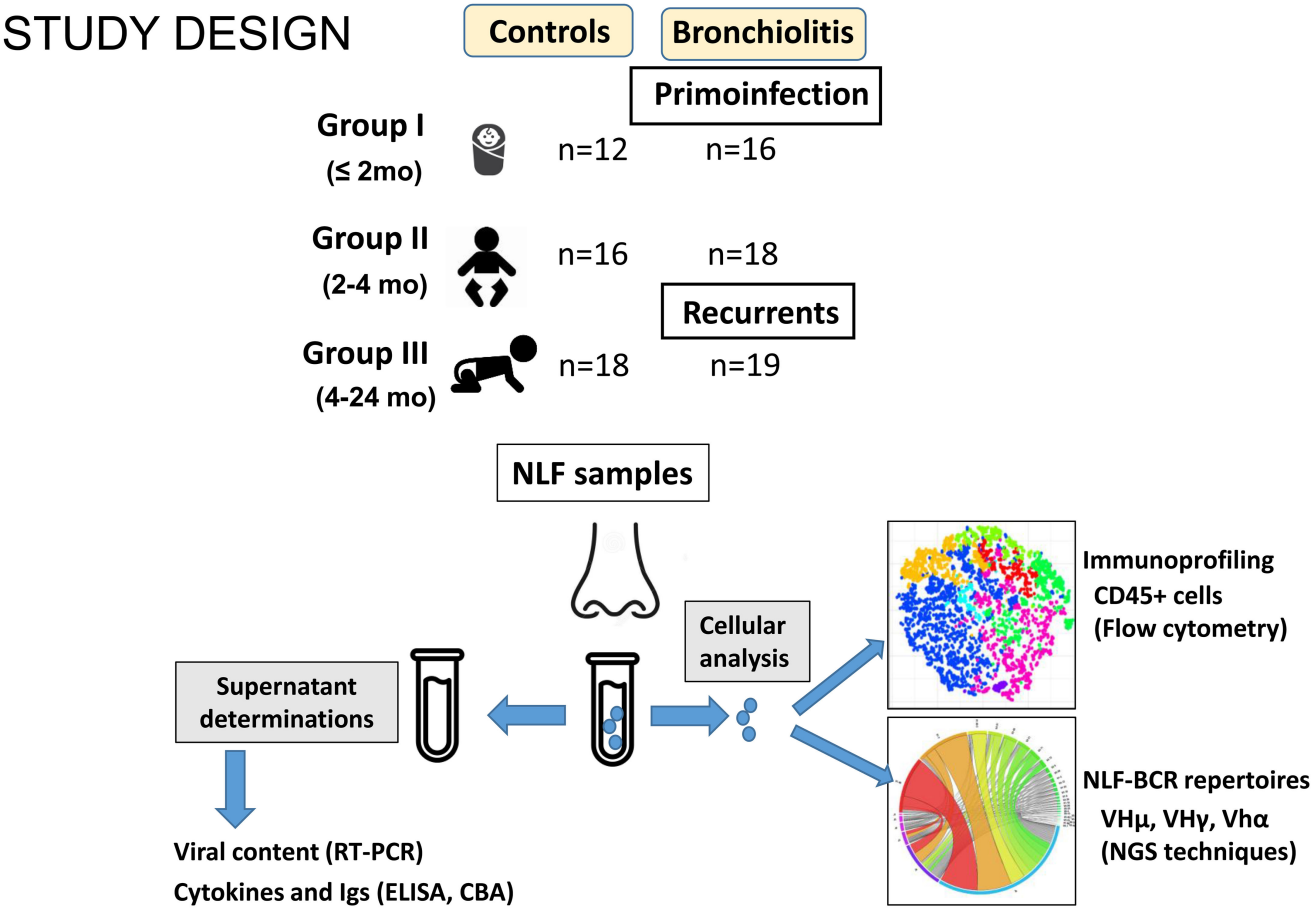
Scheme summarizing the study design. NLF samples were obtained from the indicated groups. Supernatants were used for RT-PCR, CBA and ELISA analysis to determine the viral content, cytokine profile and antibody composition, respectively. The cells recovered from NLF from bronchiolitis patients were used for immunoprofiling by using flow cytometry and for BCR IgH repertoire analyses after RNA extraction, cDNA preparation, amplification, barcoding and NGS sequencing.

NLF samples were obtained from outpatient children diagnosed with bronchiolitis (n = 53), and from a control group of children in the same age ranges (n = 46, **Table 1**). The samples were divided into three groups: GI newborns under 2-month-old, GII infants between 2-4-month-old and GIII infants between 4-24-month-old (**Figure 1**). The bronchiolitis symptoms included pulmonary distress, runny nose, cough and wheezing, and the severitycorresponded to mild or moderate pathology (20). Feeding regime of GI cohorts was different among controls (more breastfeed) and bronchiolitis, and also the GIII bronchiolitis infants, with recurrent respiratory infections, were associated with a shorter time of symptom onset. No differences were found among bronchiolitis groups in gender distribution (overall 62% males, range from 61% to 69% in GII and GIII) or in other clinical parameters, such as weight at birth, duration of gestation or vaccination protocols (**Table 1**, and not shown).

The cytokine and Ig content was ascertained in NLF supernatants from patients and controls, as well as the respiratory viruses. The immune cell content of the NLF was identified with the CD45 pan-hematopoietic marker, to distinguish hematopoietic cells, and the different immune cell populations were further characterized by flow cytometry. Cell pellets were used to prepare RNA to analyze the IgH repertoire by NGS.

The virus content results showed that 29 (54,7%) NLF from patients contained respiratory viruses, as assessed by qPCR analysis (corresponding to 56%, 38% and 68% in GI, GII and GIII respectively) and also 6 control samples (13%, **Table 1**). In agreement with previous studies, RSV was the most frequently detected virus in the patient groups, being found at a similar frequency as in other studies on outpatient samples (35), and MPV, FLU, RHINO and ADENO were also detected in some samples. By contrast, RSV, MPV and FLU were absent in all control samples analyzed.

### Increased pro-inflammatory cytokines in NLF samples in mild bronchiolitis pathology

**Figure 2:**
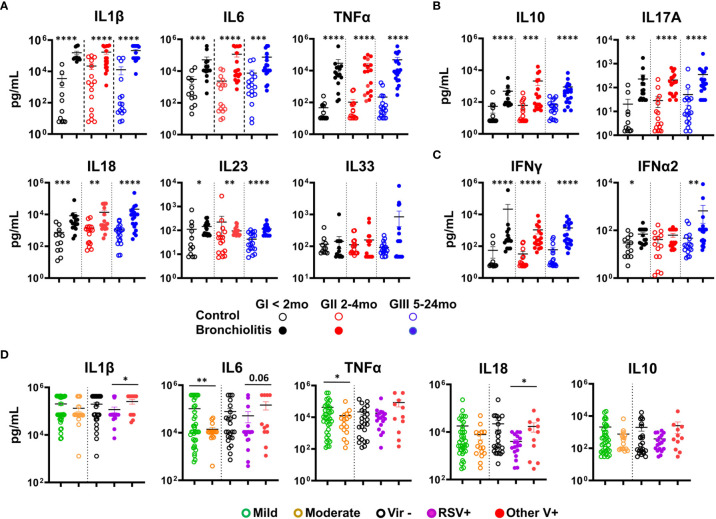
Bronchiolitis NLF samples contain elevated levels of cytokines. Cytometric bead array (CBA) determination of a panel of cytokines in the NLF supernatants from controls and bronchiolitis patients: GI (controls n = 12, bronchiolitis n = 16), GII (controls n = 16, bronchiolitis n = 18), and GIII (controls n = 18 bronchiolitis n =19). The scatter dot plots represent the individual values, and the mean ± SEM is depicted for each group. Statistical analyses were performed using non-parametric Mann-Whitney sum rank test or the unpaired t-test with Welch´s correction. **(A)** Quantification of the pro-inflammatory cytokines IL1β, IL6, TNFα, IL18, IL23 and IL33. **(B)** Determination of IL10 and IL17A, and **(C)** IFNγ and IFNα2. **(D)** Analysis of IL1β, IL6, TNFα, IL18 and IL10 in samples according to the severity of bronchiolitis (based on the Tal modified score), mild (n = 37) or moderate (n = 16) and the presence/absence of respiratory viruses: absence of virus, (Vir-, n = 24), presence of RSV (RSV+, 15 RSV alone and 3 coinfections, n = 18), presence of other virus (OtherV+, n = 11, including 9 single infections and 2 coinfections without RSV). *p < 0.05; **p < 0.01; ***p < 0.001; ****p < 0.0001.

Increased pro-inflammatory cytokines have been reported in response to RSV infection in the respiratory tract of both humans and mice (5, 7). Thus, we analyzed the cytokine profile of the NLF samples using cytometric bead arrays (CBAs). Pro-inflammatory cytokines IL1β, IL6, TNFα, IL18 and IL23 were increased in the bronchiolitis samples relative to their respective control cohort (**Figure 2A**). Interestingly, no significant differences in IL33 were detected (**Figure 2A**). There was also an augmentation in the anti-inflammatory cytokine IL10 and the regulatory IL17A (**Figure 2B**), and in IFNγ when compared with their age-matched controls. Little or no change among groups was seen for IFNα2, MCP1 or IL12p70 (**Figure 2C**, **Figure S1A** and data not shown), IL8 was very high in all samples, preventing comparisons with those from controls. In summary, bronchiolitis NLF displayed a pronounced pro-inflammatory cytokine profile, and the presence of the regulatory cytokines IL10 and IL17A, in the context of a predominant pro-inflammatory response suggests they participate in the local control of immunopathological injury (36, 37). Children with mild pattern of symptom severity displayed significant elevated levels of IL6 and TNFα (**Figure 2D**), highlighting the clinical relevance of these cytokines in these patients. When the cytokine content in NLFs were compared relative to the presence of RSV, other virus, or without detectable virus, RSV+ samples contained lower levels of IL1β, IL18 and IFNα2, as well as a tendency for IL6 and IL33 (**Figure 2D** and **Figure S1A**). In addition, no differences on cytokine production were observed among samples based on gender (not shown).

### Ig levels are higher in NLF samples from mild/moderate bronchiolitis infants

**Figure 3:**
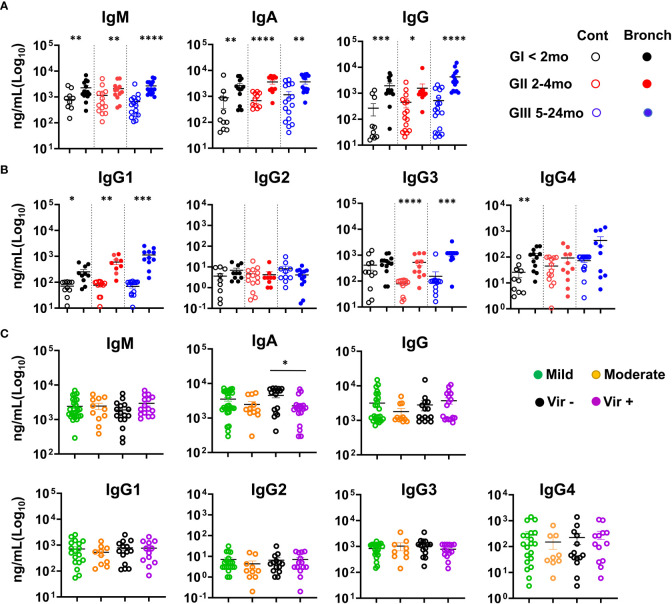
Bronchiolitis NLF samples contain elevated levels of Immunoglobulins. IgS were quantified in NLF supernatants from controls and bronchiolitis patients. The scatter dot plots represent individual values, and the mean ± SEM is depicted for each group. Statistical analyses were performed using non-parametric Mann-Whitney sum rank test. **(A)** IgM, IgA and IgG were determined by ELISA GI (controls n = 12, bronchiolitis n = 14), GII (controls n = 15, bronchiolitis n = 12), and GIII (controls n = 15 bronchiolitis n =14). **(B)** Cytometric bead array determination (CBA) for IgG1, IgG2, IgG3 and IgG4. GI (controls n = 10, bronchiolitis n = 11), GII (controls n = 14, bronchiolitis n = 9), and GIII (controls n = 14 bronchiolitis n = 10). **(C)** Ig isotype quantification based on mild (n = 29) and moderate (n = 11) bronchiolitis severity, and on the presence/absence of respiratory viruses (absence of viruses, n = 17; presence of viruses, n = 19). *p < 0.05; **p < 0.01; ***p < 0.001; ****p < 0.0001.

When IgS were measured in the NLF supernatants, IgM, IgA and IgG were detected in all the NLF samples analyzed from both patients and controls (**Figure 3A**), with higher levels in all three bronchiolitis groups than in their age-matched controls. Furthermore, increased IgG1, IgG3 and IgG4 isotypes (**Figure 3B**) were found in patient samples in comparison with their age-controls, and remained unchanged in the case of IgG2. IgA levels were higher in NLF samples from children in which no viruses were detected (**Figure 3C**). By contrast, no differences in IgM and IgG levels were found when assessed on the basis of symptom severity or of virus detection. In summary, NLF samples exhibited higher secretion of IgM, IgGs (but not IgG2) and IgA in all groups studied compared with their respective controls, and in the case of IgA, increased levels were found when not bearing detectable viruses.

### Nϕs predominate in the NLF from bronchiolitis infants

**Figure 4:**
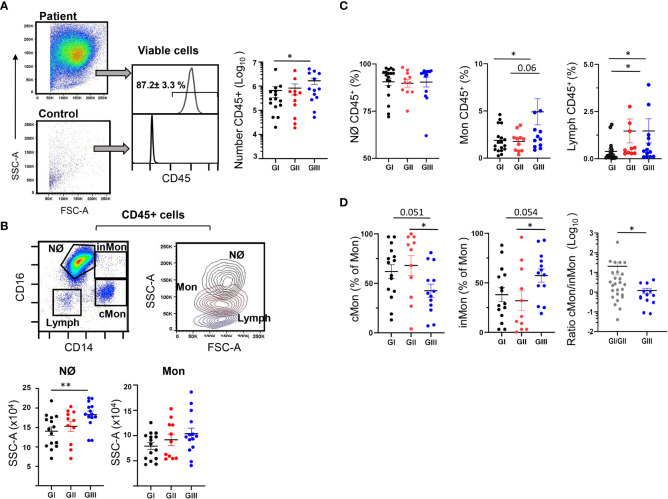
Presence of myeloid cells in NLF from bronchiolitis patients. Flow cytometry was performed on NLF bronchiolitis and control samples (n = 39 and n = 46, respectively) stained with anti-CD45, anti-CD16 and anti-CD14 MonAbs. Quantification of the myeloid cell subpopulations was performed for each group analyzed and is shown in the figure as relative (% of CD45^+^ cells). Each dot represents an individual NLF sample, also showing the mean ± SEM. **(A)** Left, representative dot plots showing the FSC-A and SSC-A in a patient sample (up) and a control (down) NLF sample after excluding the cell dump channels, doublets and dead cells (see **Figure S2A**). Middle, representative histograms of the CD45 staining to identify hematopoietic cells: Shown inside are the relative number (%, mean ± SEM, n =39) of CD45^+^ cells in bronchiolitis NLF. Control samples contained less than 10^3^ CD45^-^ cells and therefore they are not shown in the following panels. Bronchiolitis samples contained 2 x 10^6^ ± 0.6 live cells (GI), 2.73 x 10^6^ ± 0.7 live cells (GII) and 11.4 x 10^6^ ± 2.6 live cells (GIII). Right, the plot shows the quantification of CD45^+^ cells in the NLF from GI (n = 15), GII (n = 11) and GIII (n = 13). **(B)** Upper left, representative dot-plot of gated CD45^+^ cells analyzed with anti-CD14 and anti-CD16 to discriminate neutrophils (Nϕ), classical monocytes (cMon, CD14^++^CD16^-^), intermediate monocytes (inMon, CD14^++^CD16^+^) and lymphocytes (Lymph). Upper right, representative overlaid contour plot of gated CD45^+^ cells depicting the SSC-A and FSC-A analysis for neutrophils (Nϕ, black), monocytes (Mon, brown) and Lymphocytes (Lymph, blue). Bottom, determination of the SSC-A of electronically gated Nϕ and Mon in NLF samples. **(C)** Relative numbers (to CD45+ cells)of Nϕ, Mon and Lymph in each group as in panel **(B)**. **(D)** Frequency of cMon and inMon among CD45^+^ monocytes, and the cMon/inMon ratio (the latter as GI+II and GIII, n = 26 and n = 13, respectively). Comparisons were performed using non-parametric Mann-Whitney sum rank test. *p < 0.05; **p < 0.01.

We analyzed the CD45^+^ cell content of the NLF in combination with a larger panel of mAbs. The gating strategy for flow cytometry studies was optimized for NLF samples (**Figure S2A**). Less than 10^3^ cells/sample, predominantly CD45^-^, were detected in the NLF from control infants (**Figure 4A**), as described elsewhere (21). By contrast, most of the samples (38 out of 39 analyzed) from the bronchiolitis patients contained CD45^+^ cells, with a significant enrichment in the GIII NLF (**Figure 4A**). Bronchiolitis NLF contained substantial numbers of myeloid cells, mostly Nϕs (CD14^low^CD16^++^, **Figures 4B, C**), as described in BAL samples from children infected with RSV (6). There were not changes in Nϕ numbers among the groups, all of them presenting high IL8, which is the main chemokine to attract Nϕs (38). By contrast, Nϕs increased their complexity (SSC) from GI to GIII as indicative of their maturation, whereas monocytes did not (**Figure 4B** and **Figure S2C**). Since pro-neutrophil factors, such as IL17 and IL6 are elevated in NLF samples, we evaluated their relationship with Nϕ counts in the NLFs (**Figure S1B**). A correlation for IL6 and not for IL17A, and the number of Nϕs was found, which may reflect a role for IL6 in the recruitment and activationof Nϕs, as shown previously (39).

Also, monocytes were higher in infected NLF samples from GIII, and interestingly the chemotactic factor MCP1 correlated with the numbers of monocytes (**Figure S1B**). Based on their CD14 and CD16 expression, in combination with other receptors, human and mouse monocytes are defined as classical (cMon: CD14^++^CD16^-^), intermediate monocytes (inMon: CD14^++^CD16^+^) and non-classical monocytes (ncMon: CD14^low^CD16^+^), with specific inflammatory and regulatory profiles (40–42). In our analyses, as only considering CD16 and CD14 it was not possible to separate accurately ncMon from Nϕs in the NLF samples, and therefore, we focused on cMon and inMon. Frequencies of cMon were similar in the three bronchiolitis cohorts. However, inMon frequencies were higher in NLF from GIII children compared to that from GI and GII, and this led to a shift in the cMon towards inMon profile in the GIII NLF compared to GI+GII. (**Figure 4D**). Finally, the NLF Nϕ and Mon content did not differ significantly in relation to disease severity (**Figure S2D**). All these data led us to conclude that the NLF from bronchiolitis affected infants in perinatal life contains mainly Nϕs. Also, Mon were present in NFLs, in which there is a differentiation trajectory towards the inMon phenotype as the infected children age and/or suffer repetitive infections.

### Age-dependent heterogeneous T and B lymphocyte content of NLF

**Figure 5:**
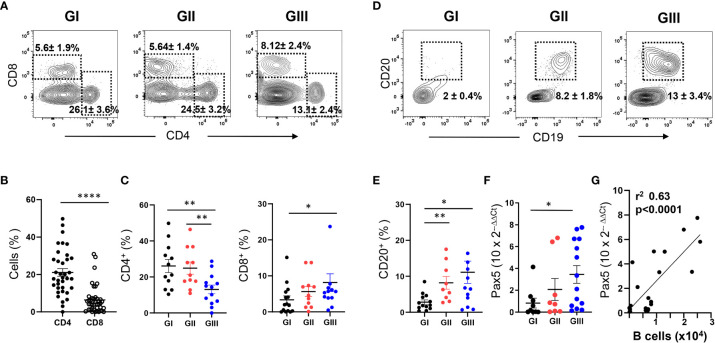
Age-dependent increase of T and B lymphocytes in NLF samples from bronchiolitis patients. Flow cytometry was performed on NLF bronchiolitis samples (n = 38) stained with anti-CD4, anti-CD8, anti-CD19 and anti-CD20 MonAbs. Quantifications were done on the lymphoid gate described in **Figure 4**. Frequency of subpopulations relative to CD45^+^ cells in this gate are shown. **(A)** Representative contour plots from GI-GIII NLF samples displaying the staining with anti-CD8 and anti-CD4 by flow cytometry. The dotted rectangles inside the plots indicate CD4^+^ and CD8^+^ T lymphocytes, and the numbers are the %, mean ± SEM of CD4^+^ and CD8^+^ cells. **(B)** Frequency of CD4^+^ and CD8^+^ cells in all NLF samples analyzed. **(C)** Frequency of CD4^+^ and CD8^+^ cells in NLF samples from GI (n = 15), GII (n = 10) and GIII (n = 13) children. **(D)** Representative contour plots displaying the staining with anti-CD20 and anti-CD19 to identify CD19^+^CD20^+^ B lymphocytes by flow cytometry. Relative numbers of B cells (%, mean ± SEM) are indicated inside the plots. **(E)** Frequency of B cell numbers in NLF samples from GI (n = 12), GII (n = 11) and GIII (n = 13). **(F)** Relative expression of PAX5 determined by RT-qPCR in NLF samples from GI (n = 9), GII (n = 8), GIII (n = 12). The amount of Pax5 transcripts was calculated as the 2^−ΔΔCT^ relative to that of GAPDH and relative to adult PBMC values, performed in duplicates. **(G)** Linear regression of Pax5 2^−ΔΔCT^ data and the number of CD20^+^ cells in NLF samples. In the graphs from panels **(B, C** and **E–G)** each dot represents an individual sample and the mean ± SEM is also shown. Comparisons among groups were done using non-parametric Mann-Whitney sum rank test. *p < 0.05; **p < 0.01; ****p < 0.0001.

A population of CD45^+^CD14^-^CD16^-^ cells with low SSC/FSC, corresponding to lymphoid cells, was also identified in the NLF (**Figure 4B**). Although the lymphoid compartment is minor compared to the myeloid component, an enrichment of lymphocytes was detected in GIII NLF samples (**Figure 4C**). As expected (43, 44), we detected both CD4^+^ and CD8^+^ T cells in the bronchiolitis NLF (**Figures 5A–C**), with overall more CD4^+^ than CD8^+^ cells (n = 33, **Figure 5B**). When analyzed according to age, percentages of CD4^+^ cells were lower in GIII samples relative to GI and GII samples and the frequency of CD8^+^ cells was higher in GIII. (**Figure 5C**).

**Figure 6:**
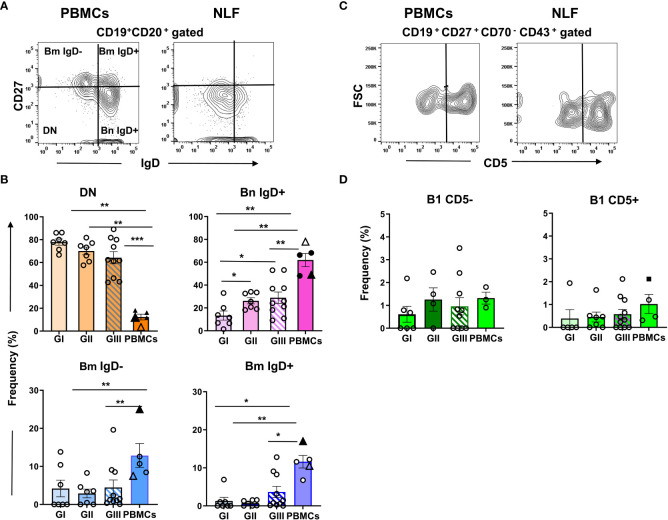
B cell phenotype in NLF from bronchiolitis affected infants. Cell suspensions were stained with the following MonAbs: anti-CD20, anti-CD19, anti-CD27, anti-IgD, anti-CD70, anti-CD43 and anti-CD5. Shown are contour plots of the indicated staining performed by using concatenated files from adult PBMC (n = 3) and from NLF samples (n = 5). **(A)** Contour plots of the CD27 and IgD staining on gated CD20^+^ B cells. The quadrants discriminate the B cell populations of naïve(IgD^+^CD27^-^ Bn), DN (IgD^-^CD27^-^) and memory (CD27^+^ Bm, IgD^-^ and IgD^+^). **(B)** Frequency of each B cell subset relative (%) to the total CD20^+^ cells on individual NLF and PBMC samples. The data are presented as scatter dot plots with the means ± SEM shown; GI (n = 7), GII (n = 7), GIII (n = 10), adult PBMC (n = 3). For comparison, the frequency obtained for adult PBMCs (*Sanz et al.*, filled triangle) and that of PBMCs from 1-24 mo-old children (*Berrón-Ruiz et al.*, empty triangle) are shown. **(C)** Contour plots representing CD5 versus FSC-A on gated CD19^+^CD27^+^CD70^-^CD43^+^ B1 cells. **(D)** Frequency of CD5^-^ and CD5^+^ B1 cell subsets as the % of the total CD19^+^ cells. Filled square shows the frequency obtained on adult PBMCs (*Rodriguez-Zhurbenko et al.).* Data are represented as in **(B)**; GI (n = 6), GII (n = 7), GIII (n = 11), PBMC (n = 3). Data were compared using non-parametric Mann-Whitney sum rank test. *p < 0.05; **p < 0.01; ***p < 0.001.

Few CD20^+^ B cells were found in GI NLF, whereas their number augmented in the NLF from GII and GIII infants (**Figures 5D, E**). The presence of B cells in these NLF samples was confirmed by Pax5 transcription factor detection, the levels of which correlated with the number of B cells (**Figures 5F, G**). The B cell composition of the NLF was analyzed (22), revealing the presence of different B cell populations in NLF samples (**Figure 6**). CD19^+^CD20^+^ cells negative for IgD and CD27 (DN cells) and IgD^+^ naïve B cells predominate. These DN B cells have been reported as atypical memory or tissue-based memory cells, that may follow an extrafollicular differentiation pathway (22). Only small fractions of CD27^+^ memory B cells and also very few CD27^+^CD70^-^CD43^+^CD5^+/-^ B1 cells (**Figures 6C, D**) were found, these latter at similar numbers than those described for peripheral blood mononuclear cells (PBMC) from children under 2-years-old (45, 46) and adults (47).

From all these data we conclude that in the NLF of bronchiolitis children there is an age-dependent variation in the lymphoid compartment, containing CD4^+^ and CD8^+^ T cells from neonatal life, and distinct B cell subsets in infants older than 2 mo of age.

### IgH repertoires with low mutation rates in NLF

**Figure 7:**
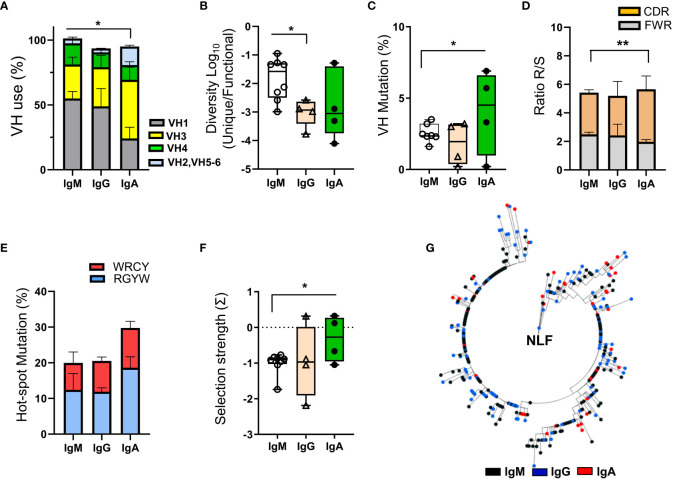
BCR repertoire analysis in NLF. The NLF repertoires of VDJ- IgM (n = 9), -IgG (n = 4) and IgA (n = 4) rearrangements were determined after RT-PCR-specific amplifications and sequencing by NGS. The obtained sequences were processed, cleaned and analyzed as indicated in Materials and Methods (“Bioinformatics” section). **(A)** VH usage frequency on sequences from each individual repertoire of NLF samples. Shown are the means ± SEM. See **Table S2** for sequence numbers. **(B)** The diversity of the repertoires was calculated as the ratio between unique sequences and functional sequences. Each dot represents the value for the repertoire of all sequences obtained in the same sample, and overlaid are box-and-whisker plots showing the median, the first quartile to the third quartile and the minimum and maximum values. **(C)** VH mutations in IgS repertoires. Data are represented as in **(B)**. **(D)** The ratio of replacement (R) and silent (S) mutations located in the CDRs and FWRs is shown as mean ± SEM. **(E)** Frequency of SHM hotspots in the AID motifs WRCY and RGYW. Data are shown as mean ± SEM. **(F)** Antigen selection strength (quantified using the BASELINe algorithm). Data are represented as in **(B)**. **(G)** Clonal tree obtained for the VH3-23-JH4 family clone identifying IgM (n = 198), IgG (n = 113) and IgA (n = 46) sequences, from a single NLF sample. The analysis was performed based on a minimal substitution model using MUSCLE software, with alignment curation using Gblocks and tree interference with PhyML. Data were compared using an unpaired two tailed Student´s t-test. *p < 0.05; **p < 0.01.

RT-PCR amplification of isotype-specific IgH was performed on cell pellets obtained from GII/GIII NLF, yielding IgM amplification in 69% of the samples analyzed, and in 38% each of IgG or IgA amplifications (data not shown). The IgH repertoire was analyzed in these samples (**Figure 7** and see **Table S2**) and not in these from NLF samples healthy donors, because they did not contain CD45^+^ cells. Productive IgM-repertoires from NLF-samples showed a higher usage of the VH1 family with respect to IgA-repertoires, that had higher number of VH3 sequences (**Figure 7A** and **Figure S3**).

The CDR3 length of the NLF repertoires of IgM, IgG and IgA was similar (**Figure S3D**). Despite there was low sequence diversity among the individual repertoires (**Figure 7B**), those corresponding to IgM-sequences were higher, as confirmed by the Shannon entropy index (**Figure S3E**). In terms of VH mutations and R/S ratios (**Figures 7C, D**), IgM-repertoires displayed lower mutations in VH regions and in AID targeted hotspot RGYW motifs than IgA-repertoires (**Figure 7E**). In this sense, antigen driven-selection, determined using the BASELINe algorithm, showed that IgA sequences from NLF underwent positive antigenic selection (**Figure 7F**, **Figure S3F**). Finally, a clonal analysis tree performed in one sample (NLF62, **Table S2**) for VH3-23.JH4, one of the most abundant clones in human samples (25), showed intraclonal Ig switching (**Figure 7G**).

In summary, these results show the preferential usage of VH1 in IgM and IgG sequences of NLF bronchiolitis infants, with little diversity and mild selection strength, except for IgA sequences. Furthermore, intraclonal switching can be detected, suggesting specific selection processes in mucosal territories from early stages of infancy in the context of respiratory infections.

## Discussion

**Figure 8:**
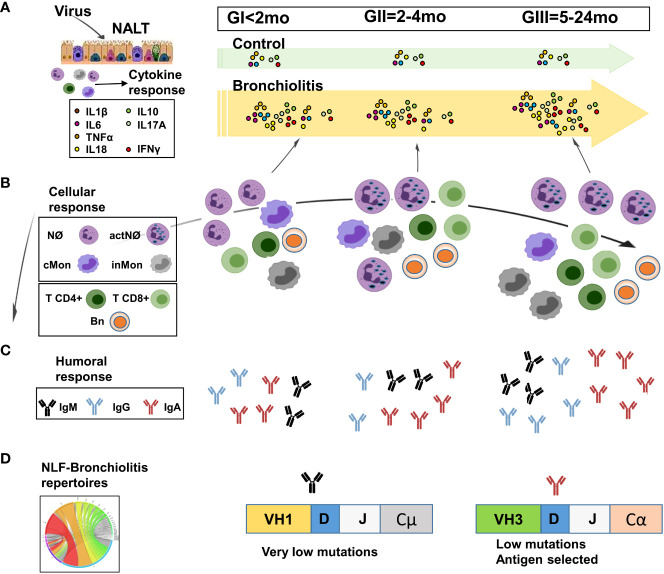
Scheme of the proposed age-dependent nasopharynx-associated lymphoid tissue (NALT) responses in children with mild/moderate bronchiolitis pathology. The cytokine, immunoglobulin and cellular content in NLF samples varies with the age of the children affected with bronchiolitis. **(A)** Pro-inflammatory and regulatory cytokines are present in all NLF bronchiolitis samples at higher levels than in these from controls. **(B)** Heterogeneous combinations of CD45^+^ innate and adaptive cell types are detected in NLF from patients and not in controls. Neutrophils (Nϕs) are predominant in all NLF samples groups, which also contain classical and intermediate monocytes (cMon and inMon) and lymphocytes, these two latter increasing in children older than 2 months (GII/GIII), in which activated Nϕs (actNϕs) with increased complexity and cMon and inMon, T cells (CD4^+^ and CD8^+^) and CD19^+^ cells were found. **(C)** IgM, IgGs and IgA levels are present at higher amounts than in age-matched controls. In addition, IgG levels highly augment in GIII infants. **(D)** Local B cell repertoires were determined by analyzing the presence of VH rearrangements in NLF samples, which displayed IgA- sequences with low mutations but antigen selection in GII/GIII infants. Created with BioRender.

This study presents an age-stratified analysis of nasal mucosal immunity in bronchiolitis infants, having collected NLF from children suffering their first infection and older infants who have experienced repetitive respiratory infections. Perinatal life and early infancy is a period of enhanced susceptibility to pathogens due to the immaturity of immune responses (48). The neonatal environment favors the down regulation of Th1 cytokine responses, with regulatory cytokines such as IL10 and IL17 following a developmental trajectory (49). These regulatory cytokines promote a Th2-profile and they are released by different cellular compartments, including subsets of myeloid and lymphoid cells (50, 51). In our study there was an important increase in pro-inflammatory cytokines (IL1β, IL6, TNFα, IL18, IL23) upon infection in all the groups analyzed in comparison with age-related control samples, as also seen for the immunoregulatory cytokines IL10 and IL17A, and for Th1 IFNγ NLF (**Figure 8A**). IL17 plays a pivotal role in neutrophil responses (52, 53). However, no significant correlation has been found between IL17A and the number of Nϕs in bronchiolitis NLF samples, at difference to the positive correlation found for IL6, which has been reported to have a dual role (pro-inflammatory and anti-inflammatory) (54, 55). In NLF from infants hospitalized with RSV bronchiolitis, elevated IL33, among other Th2 cytokines and low levels of IFNγ, was detected in the acute phase of the disease (19, 56–58), predisposing them to allergic inflammation and favoring eosinophilia. In contrast, our data showed no increased levels of IL33 in mild/moderate infants. Interestingly, some cytokines, including IL6 and TNFα (but not IL33), were significantly enriched in patients with a mild pathology. It is possible that these cytokines reflect the engagement of an early innate immune response able to effectively resolve the pathology and thus leading to a mild symptomatology. In this sense, it has been proposed that elevated levels of IL6, IFNγ and IL10 among others, protect against hypoxia in bronchiolitis (59). One of the limitations of the present study is that we have focused on outpatient samples, and therefore there is a lack of hospitalized patients with a severe pathology. We believe that the first antigenic encounters in the respiratory mucosa might have an impact on future respiratory infections in these cohorts, perhaps with a different profile than in hospitalized children with severe pathology. In this sense, it will be interesting to determine whether the children with mild/moderate bronchiolitis are more/less prone to the development of asthma, in comparison with children with severe bronchiolitis. Also, it will be interesting to study NLF adult samples to address the adult NALT immune responses to respiratory viruses. Another limitation of this study relays on the low presence of RSV in the NLF bronchiolitis samples. RSV has tropism to higher and lower airways yielding mild symptoms in the upper tract as opposed to severe in lower respiratory tract. The low detection of virus that we found in NLF could be related to the fact that we have collected these upper respiratory samples in the first 24-48hr of infection, making it difficult to detect the virus in the onset of mild/moderate pathology. On the other side, NLF from most of bronchiolitis diagnosed patients had CD45^+^ cells, but none was found in the healthy control samples, regardless the presence in these samples of other viruses different from RSV, MPV, or FLU. This finding led us to propose tracing CD45^+^ in NLF samples as an indicator of NALT-immune responses associated with bronchiolitis pathology. However, it is possible that age dependent cellular composition changes may contribute to our results, since the lack of CD45^+^ cells in the age-matched controls avoided comparisons with them.

Nasopharynx-associated lymphoid tissue (NALT) is related with the induction of mucosal immune responses (60), and the activation of NALT that occurs in mice after birth is regulated by the presence of environmental antigens and mitogens (61). The data presented here demonstrate the age-dependent recruitment of distinct effector immune cells (innate and adaptive cell populations) in NLF from infants affected by bronchiolitis, indicative of NALT activation as a consequence of respiratory infection (**Figure 8B**). We detected a pronounced Nϕ accumulation in all bronchiolitis NLF samples, as described for BAL samples (62). This accumulation has been related to the antiviral action of Nϕs, involving the release of antimicrobial peptides, elastase, myeloperoxidase and increased phagocytosis (8). Immature granulocytes lacking pro-inflammatory proteins in their granules are abundant in neonates (63), which are more vulnerable to infection. Interestingly, Nϕ complexity in the NLF from children increased with age in the context of respiratory infections, as described (64). Also, cMon were present in all the samples analyzed, although there were more inMon in the NLF from GIII infants, a fact that might reflect the regulatory mechanisms implemented to overcome the repetitive infections that define this group of children. Secretion of IL6 and IL10 by differentiated macrophages has been described in immunosuppressive tumor-associated -macrophages, rheumatoid arthritis, asthma and regulating epithelial integrity in the small intestine (65). Thus, it may be feasible that differentiated macrophages present in the GIII group have an immunoregulatory role in the respiratory mucosa where there are higher numbers of monocytes.

Adaptive immune responses mediated by T cells were evident in the newborn samples (GI) and they were maintained over time. Depletion of CD4^+^ T cells in neonatal RSV infected mice minimally affected the humoral responses (66) and TF_H_ and antibody responses are low in early life in mouse models of RSV (67), indicating that neonatal antibody responses against RSV are mainly T cell independent (66). Several studies of the specific-adaptive immune response against RSV were performed using a formalin-inactivated RSV (FI-RSV) vaccine, which failed in human trials because of the exacerbation of the disease after subsequent natural infection (68). FI-RSV immunized mice elicited a mixed Th1 and Th2 CD4^+^ cytokine response (69) and FI-RSV vaccination of mice accelerated the formation of primary lymphoid nodes, with delayed B cell responses especially for the Ab isotype switch (70). Interestingly, a proportion of the CD45^+^ lymphoid cells were not T or B cells, and thus they might correspond to other cell types, including NK cells Tγδ CD4^-^CD8^-^ cells, and innate lymphoid cells (ILCs). In this sense, ILC2 cells (CD45^+^lin-ST2^+^c-kit^+^Sca-1^+^) have been reported in nasal aspirates from children and in the lungs of RSV infected neonatal mice with severe RSV-immunopathogenesis, in association with higher levels of IL33 (50, 71).

B cells were detected in NLF from 2 mo of age (GII and GIII) but not so readily before that age. A detailed analysis of the B cell compartment in the NLF samples highlighted a diverse composition, including unswitched and switched naïve cells accompanied by minor numbers of other B cell populations **(** **Figure 8B**). These results agree with the major naïve B cell subset, together with an early presence of memory and plasma cells, in peripheral blood from infants under 24 month-old (45, 46). Thus, the detection of B lymphocytes in NLF from 2-mo-old infants may reflect a local immature adaptive humoral response independent of the canonical germinal center responses configured after 1 year of age (72, 73).

The finding that control children without a respiratory pathology have detectable amounts of IgS in their NLF (this study and (74, 75) is aligned with the notion that in a homeostatic state, mucosal barriers have a protective layer of soluble factors, including IgS produced by local cells, as described for the lamina propria (76, 77). We found a large increase in levels of IgM, IgGs (but not in the case of IgG2) and IgA upon infection in NLF from all the groups analyzed in comparison with their age-related controls (**Figure 8C**). Interestingly, IgA levels in NLF samples containing viruses were lower than in those without them. It may be possible that this effect is due to IgA binding to the respiratory viruses. Also, there was higher IgG and IgA antibody content in NLF from bronchiolitis children relative to their age-control cohorts. Since IgG2 has low placental transport (78), this may explain the low levels of IgG2 found in NLF. Indeed, neonates rely heavily on passive maternal Ab transfer for protection against infections (79), and maternal IgGs downregulate B cell maturation (80). We cannot rule out the contribution of maternal IgGs and IgA on the ELISA quantitation in NLF samples. However, the fact that the same preparations contained B cells with detectable IgG- and IgA-BCR repertoires, points to an active participation of the neonatal humoral response in the IgG and IgA antibody NLF content, prompting us to analyze their NLF-IgH-repertoire. This analysis indicated: (i) the presence of productive IgH rearrangements starting at 2-month old bronchiolitis infants; (ii) a dominant VH1 usage in the IgM and IgG repertoires and VH3 in IgA sequences; (iii) more diverse CDRs in IgM-repertoires than in IgG; (iv) higher VH mutation and selection strength in IgA-repertoires, and (v) intraclonal switching among VH family sequences in the NLF. In agreement with our results, it was described a predominant usage of VH1 (–2, –18, –69), VH2-70, VH4-04 and VH5-51 in PBMCs from RSV infected adult patients (81). A bias towards the VH1-46 rearrangement has also been described in response to rotavirus (82) and VH1-69 has been preferentially found in response to HIV (83). By contrast a preferential use of VH3 and VH4, was detected in neonatal cord blood and healthy Ad-PBMC (18), indicating that the IgH-repertoires of NLF sequences differ from those of healthy PBMC-repertoires (84). Switched IgG- and IgA-adaptive responses at mucosal surfaces require the contribution of the germinal center, although in some cases human IgA mucosal responses may develop without T cell help (85). Our data regarding IgA-repertoires in NLF suggest the potential of the NALT to develop local selected antigenic responses in the context of infant respiratory infections before the advent of canonical germinal center responses (**Figure 8D**). Future experiments in this line are needed in order to reinforce these findings, with an increased number of NALT-repertoires and their direct comparison with PBMC-repertoires from the same cohort.

Together, our results represent an integrated humoral, cellular and BCR-repertoire characterization of the NLF from infant bronchiolitis patients that is not only relevant to this pathologic disorder but also, to other respiratory infections (such as COVID-19, pneumonias or COPD) and to different age groups. The management of acute bronchiolitis in infants remains a clinical challenge for pediatricians due to its lack of response to any current available medication, as well as its high morbidity. Taken together, our results highlight the importance of NALT immune responses in newborns and young infants with bronchiolitis, using NLF as non-invasive biological samples. We believe that a better understanding of the NALT immune response may improve the development of potential therapies that could modify the clinical course of these patients, who are in full immune maturation throughout the first year of life.

## Data availability statement

The original contributions presented in the study are included in the article/**Supplementary Material**. Further inquiries can be directed to the corresponding authors.

## Ethics statement

The studies involving human participants were reviewed and approved by ethics committee of Gerencia Asistencial de Atención Primaria (#14/17) and that of the Hospital 12 de Octubre (#15/355). Written informed consent to participate in this study was provided by the participants’ legal guardian/next of kin.

## Author contributions

IC performed experiments, analyzed the data, and interpreted the results. MR and AA performed experiments and analyzed the data. SH, CG-V, JR, SF, JD, BR, BS, CA, and FG, collected samples and clinical information. AZ performed the NGS experiments. SR performed the t-SNE analysis and discussed the results. VL performed the bioinformatics analyses. M-LG and BA directed the project, provided guidance for the research and wrote the manuscript. All authors contributed to the article and approved the submitted version.
