# Supplementary material for: Age-dependent nasal immune responses in non-hospitalized bronchiolitis children
Source: Front Immunol. 2022 Dec 6;13:1011607. doi: 10.3389/fimmu.2022.1011607 (PMC9763932; doi:10.3389/fimmu.2022.1011607)
Supplement: Supplementary file 1 [file DataSheet_1.pdf]

Supplementary Information

"Age-dependent nasal immune responses in non-hospitalized bronchiolitis children."

I. Cortegano, M. Rodríguez, S. Hernángómez, A. Arrabal. C. Garcia-Vao, J. Rodríguez, S. Fernández, J. Díaz, Belén de la Rosa, Beatriz Solís, Cristina Arribas, Felipe Garrido, A. Zaballos, S. Roa, V. López, M.L. Gaspar*, B. de Andrés*.

*****Correspondence: Belén de Andrés or Maria Luisa Gaspar bdandres@isciii.es; mlgaspar@isciii.es.

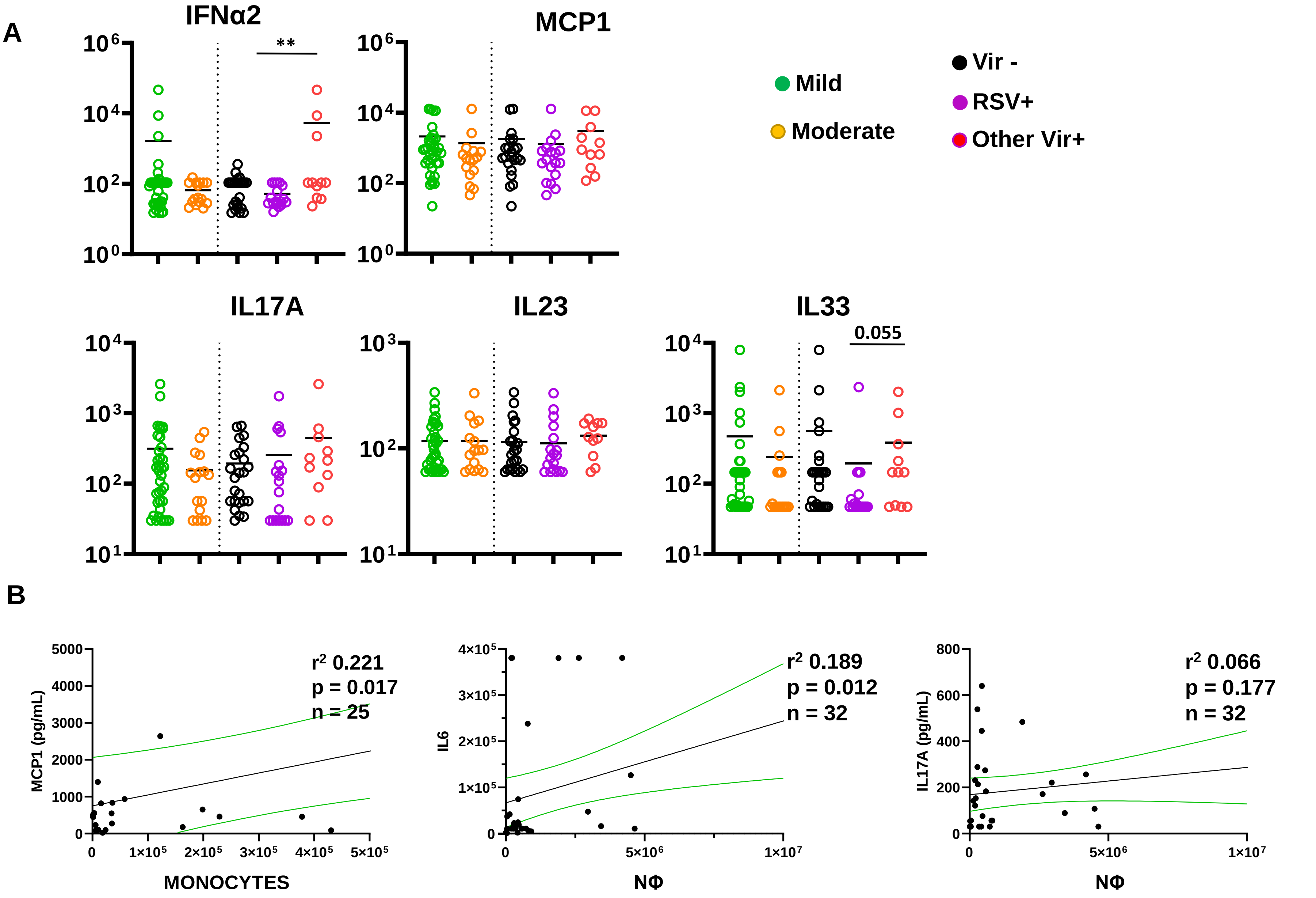

Figure S1: Cytometric bead array (CBA) and regression curves. **(A)** Quantification analysis of IFNα2, MCP1, IL17A. IL23 in brinchiolitis NLF samples according to the severity of bronchiolitis (based on the Tal modified score), mild (n = 37) or moderate (n = 16) and absence of viruses (n = 24), presence of RSV (n = 18), and presence of other virues (n = 11). Statistical analyses were performed using non-parametric Mann-Whitney sum rank test or the unpaired t-test with Welch´s correction. **(B)** Curve regression analysis between the number of monocytes and MCP1 (left), the number of granulocyes and IL6 (middle) and the number of granulocytes and IL17A (right). **p<0.01.

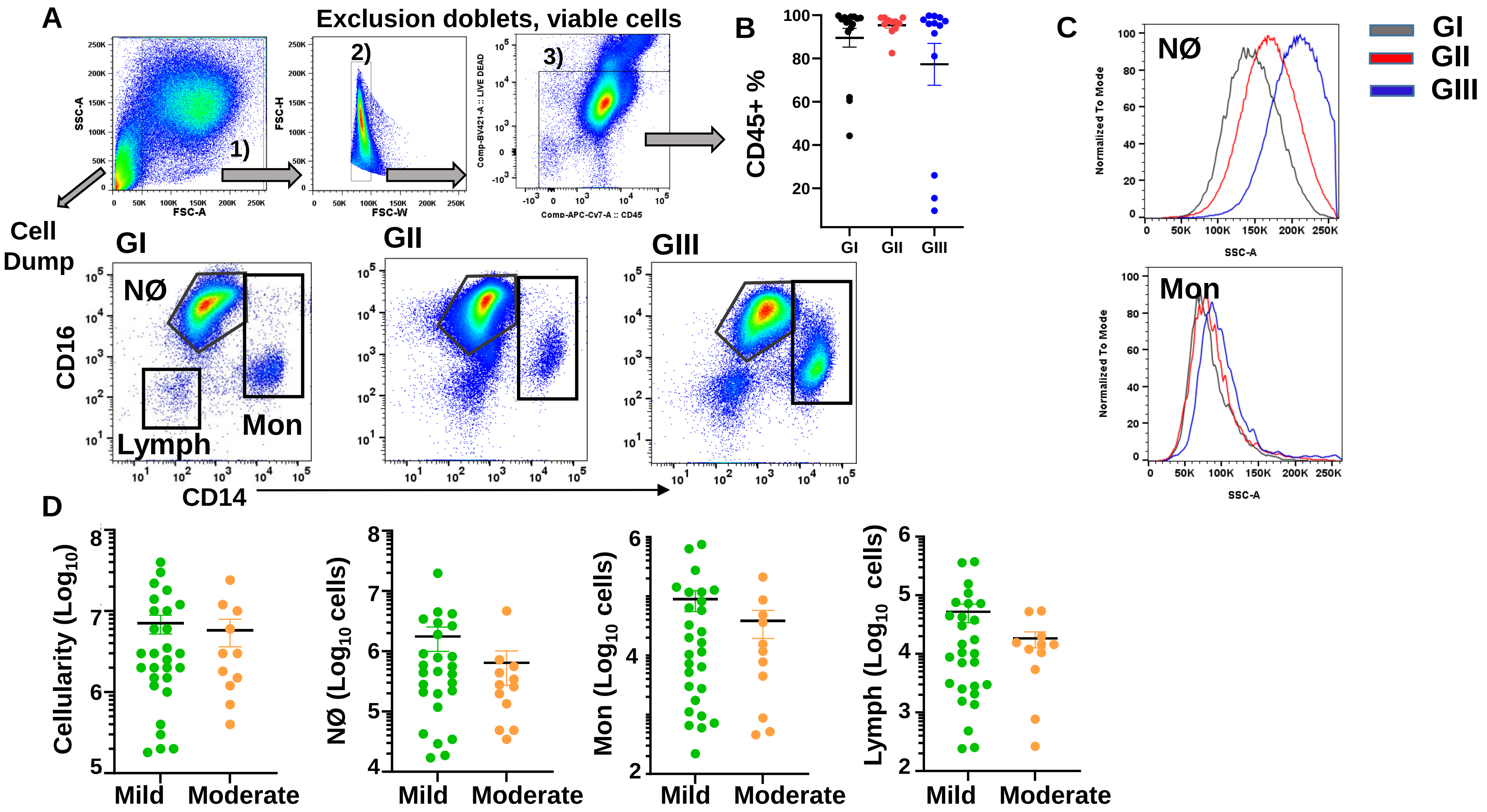
A
Exclusion doblets, viable cells
1)
2)
3)
Cell Dump
SSC-A
FSC-A
FSC-H
FSC-W
Comp-BV421-A :: LIVE DEAD
Comp-APC-Cy7-A :: CD45
B
CD45+ %
GI
GII
GIII
C
NØ
Mon
Normalized To Mode
SSC-A
GI
GII
GIII
NØ
Mon
Lymph
CD16
CD14
D
Cellularity (Log10)
NØ (Log10 cells)
Mon (Log10 cells)
Lymph (Log10 cells)
Mild
Moderate

Figure S2: Flow cytometry gating strategy on NLF bronchiolitis samples and quantitation of celular populations. (A) NLF samples were stained with anti-CD45, anti-CD16 and anti CD14 in order to be analyzed in a Fortessa cytometer. Shown are dot plots of a representative NLF sample and the gating strategy to exclude (up) 1) debris (SSC-A vs FSC-A), 2) doblets (FSC-H vs FSC-W) and 3) dead cells (Live/Dead BV421 vs CD45-APC.Cy7), and the CD14 vs CD16 staining to discriminate neutrophils (Nϕ, CD14-CD16+), monocytes (CD14+) and lymphocytes (CD14-CD16-) (Bottom). **(B)** Quantitation of CD45+% cells on each group, GI (n = 15), GII (n = 11), and GIII (n = 13). **(C)** The overlaid histograms for SSC-A values of gated Nϕ and Mon from GI, GII and GIII. **(D)** Analysis of the severity (Mild/Moderate) and the cellular content in NLF samples. Calculations of the numbers were performed as described in Figure 4. Cellularity of the samples and numbers of NØ, Mon and Lymph were determined. Mild (n = 28), Moderate (n = 11). Each dot in panels (B and D) represents an individual NLF sample, also showing the mean ± SEM. The data were compared among the three groups using non parametric Mann-Whitney sum rank test between groups.

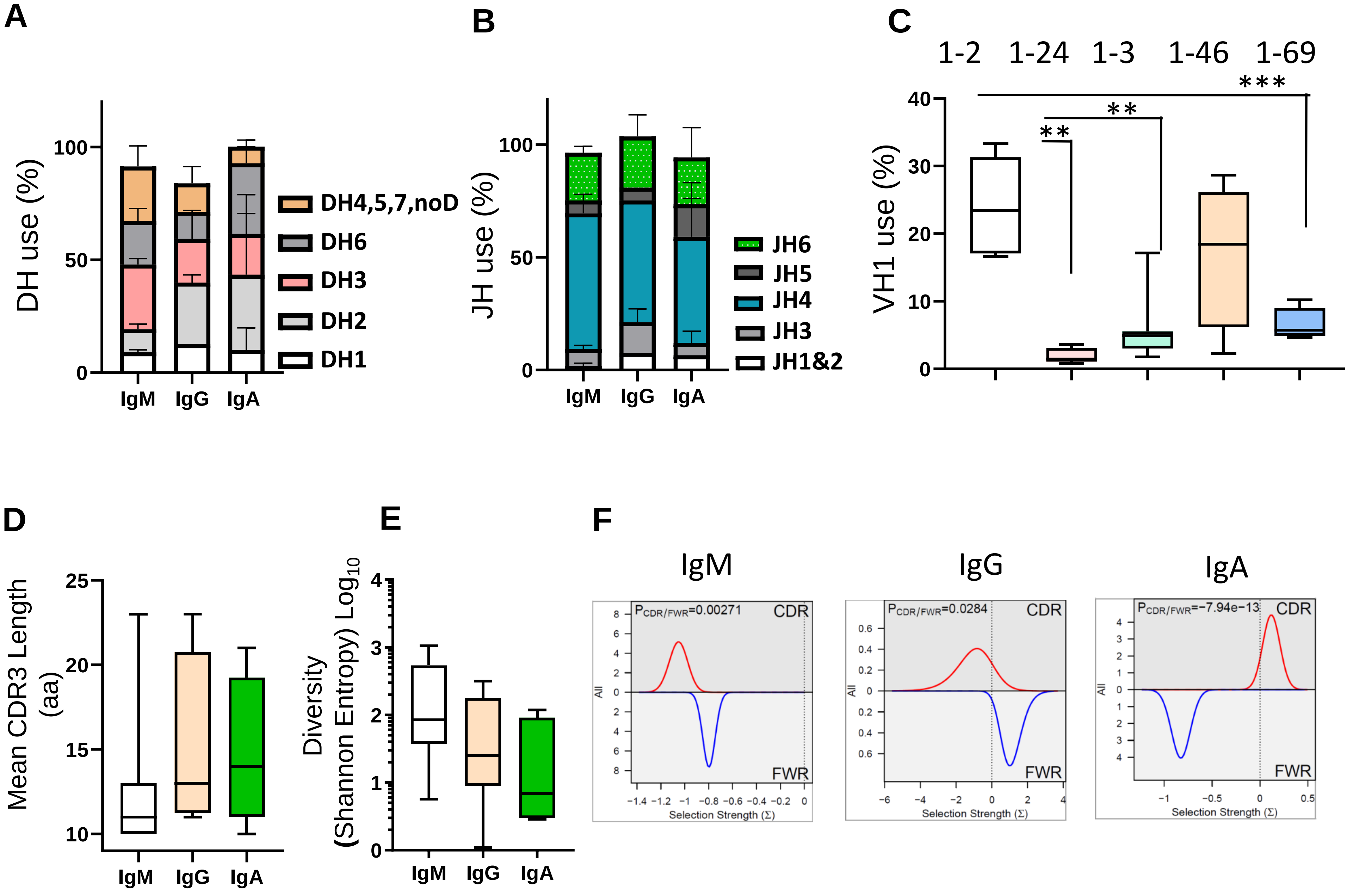
A
DH use (%)
100
50
0
IgM
IgG
IgA
DH4,5,7,noD
DH6
DH3
DH2
DH1
B
JH use (%)
100
50
0
IgM
IgG
IgA
JH6
JH5
JH4
JH3
JH1&2
C
1-2
1-24
1-3
1-46
1-69
***
**
**
VH1 use (%)
40
30
20
10
0
D
Mean CDR3 Length (aa)
25
20
15
10
IgM
IgG
IgA
E
Diversity (Shannon Entropy) Log10
4
3
2
1
0
IgM
IgG
IgA
F
IgM
IgG
IgA
PCDR/FWR=0.00271
PCDR/FWR=0.0284
PCDR/FWR=−7.94e−13
CDR
FWR
All
Selection Strength (Σ)

Figure S3: Immunoglobulin repertoire analysis of NLF samples. The sequences generated by NGS, were processed, cleaned and analyzed as indicated in detail in the Bioinformatics section (Materials and Methods), and are expressed as repertoires for each sample. IgM, n = 9; IgG, n = 4; IgA, n = 4. VH1, DH and JH frequencies were obtained from the bioinformatic tool ARGalaxy. **(A)** Immunoglobulin-DH used in the rearrangements expressed as frequencies. Data are means ± SEM. **(B)** Immunoglobulin-JH used in the rearrangements expressed as frequencies. Data are means ± SEM. **(C)** More abundant rearrangements of the IgM VH-1 family found in IgM-NLF repertoires expressed as frequencies. Data are presented as box-and-whisker plots showing the median, indicating the first quartile to the third quartile and the minimum and maximum values. **(D)** CDR3 mean length (aa) for IgM, IgG and IgA sequences from individual repertoires. **(E)** The diversity of the repertoires was calculated using the Shannon entropy of CDR3 variability. The data in panels **D** and **E** are presented as box-and-whisker plots showing the median, indicating the first quartile to the third quartile and the minimum and maximum values. Comparisons among groups were performed using non-parametric Mann-Whitney sum rank test. **(F)** Selection strength representative diagrams of NLF repertoires using the BASELINe algorithm. **p<0.01; ***p<0.001.

Table S1: List of primers used

Gene	Primer 5´	Primer 3´	PCR fragment (bp)	Temp anneal	Reference
GAPDH	GAGTCAACGGATTTGGTCGT	TTGATTTTGGAGGGATCTCG	237	60ºC	*This study*
PAX5	CTGGACAGGGCAGCTACTCAG	CTGAGGGTGGCTGTAGGGACT	85	60ºC	*Ray et al*
MID tags	TCGTCGGCAGCGTCAGATGTGTATAAGAGACAG	GTCTCGTGGGCTCGGAGATGTGTATAAGAGACAG			*Wu et al*
IgVH	MIX:<br>MID-CCTCAGTGAAGGTCTCCTGCAAGG<br>MID-TCCTCGGCTGGTGAAACCCACACA<br>MID-GGTCCCTGAGACTCTCCTGTGCA<br>MID-TCGGAGACCCTGTCCCTCACCTGC<br>MID-CAGTCTGGAGCAGAGGTGAAA<br>MID-CCTGTGCCATCTCCGGGGACAGTG	IgM MID-GGGGAATTCTCACAGGAGAC<br>IgG MID-GAGTTCCACGACACCGTCAC<br>IgA MID-GGCTCCTGGGGGAAGAAGCC	350-400<br>400-450<br>400-450	58ºC	*Wu et al*

-Ray D, Kwon SY, Tagoh H, Heidenreich O, Ptasinska A, Bonifer C. Lineage-inappropriate PAX5 expression in t(8;21) acute myeloid leukemia requires signaling-mediated abrogation of polycomb repression. Blood. 2013 Aug 1;122(5):759-69. doi: 10.1182/blood-2013-02-482497. Epub 2013 Apr 24. PubMed PMID: 23616623.

- Wu YC, Kipling D, Leong HS, Martin V, Ademokun AA, Dunn-Walters DK. High-throughput immunoglobulin repertoire analysis distinguishes between human IgM memory and switched memory B-cell populations. Blood. 2010 Aug 19;116(7):1070-8. doi: 10.1182/blood-2010-03-275859. Epub 2010 May 10. PMID: 20457872; PMCID: PMC2938129.

Table S2

Summary of IgH NGS results					
			**IgM**	**IgG**	**IgA**
**Total productive sequences**		**Number B cells/mL (CD19+CD20+)**	125053	70040	62203
**Group II**	**#NLF9**	220	143	7*	0
	**#NLF14**	168	78	0	0
	**#NLF62**	120000	30763	37030	8009
	**#NLFA104**	ND	35	23	3*
**Group III**	**#NLF10**	990	15	4*	0
	**#NLF11**	100	41	24	35
	**#NLF13**	7800	15	0	0
	**#NLF25**	68000	2148	3*	22
	**#NLF53**	3720	49	260	298
**TOTAL NLF**			**33287**	**37350**	**8377**

ND, not done

*Samples <15 productive sequences/patient were not used for repertoire analysis.
